# Merging High-Throughput, Amplicon-Based Second and Third Generation Sequencing Data: An Integrative and Modular Data Analysis Framework for Haplotype Prediction and Output Evaluation

**DOI:** 10.3390/ijms26073443

**Published:** 2025-04-07

**Authors:** Sylvia Mink, Christian Attenberger, Yannik Busch, Johanna Kiefer, Wolfgang Peter, Janne Cadamuro, Tim A. Steiert, Andre Franke, Christoph Gassner

**Affiliations:** 1 Central Medical Laboratories, Carinagasse 41, 6800 Feldkirch, Austria; 2 Institute of Translational Medicine, Private University in the Principality of Liechtenstein, 9495 Triesen, Liechtenstein; 3 Faculty of Medical Science, Private University in the Principality of Liechtenstein, 9495 Triesen, Liechtenstein; 4 Stefan-Morsch-Stiftung, 55765 Birkenfeld, Germany; 5 Institute for Transfusion Medicine, Faculty of Medicine and University Hospital Cologne, University of Cologne, 50923 Cologne, Germany; 6 Department of Laboratory Medicine, Paracelsus Medical University Salzburg, 5020 Salzburg, Austria; 7 Institute of Clinical Molecular Biology, Christian-Albrechts-University and University Medical Center Schleswig-Holstein, 24118 Kiel, Germany

**Keywords:** next-generation sequencing, third generation sequencing, raw data, haplotypes, phasing, amplicon-based, ONT, Illumina, data analysis framework

## Abstract

Despite providing highly accurate results, the short reads generated by second generation sequencing have major limitations in mapping complex genomic regions. Longer reads can resolve these issues and additionally phase distant variants. The third generation sequencing platform ONT currently achieves the longest sequencing reads but falls short in sequencing accuracy. Additionally, deriving phased haplotypes from amplicon-based NGS data remains a complex and time-consuming task that requires extensive bioinformatic expertise. We constructed an integrative, open-access modular data-analysis framework that allows for automated processing of high-throughput sequencing data from both second (Illumina) and third generation (ONT) sequencing platforms, combining the strengths of both technologies. Variant information is automatically evaluated and color-coded for discrepancies. Haplotypes are listed by frequency. All parts of the framework can be used independently. The framework’s performance was validated using synthetic and tested with real-life data by analyzing partly homologous *FUT1*/*2*/*3* sequencing data from 400 blood donors.

## 1. Introduction

Second generation, short-read, massively parallel sequencing and the latest third generation long-read, single-molecule sequencing technologies have revolutionized the field of genomics by generating extensive amounts of genomic data in relatively short turnaround times [1,2].

The Illumina second generation sequencing platform is renowned for generating highly accurate sequencing reads (>99.9%), making it the gold standard of clinical and research sequencing [1,3]. However, a notable drawback is that the platform typically produces short paired-end reads of up to 1000 bp [4], which imposes several important limitations.

A relevant percentage [5] of the human genome consists of duplicate, highly homologous, or highly repetitive sequences to which short reads either cannot be uniquely mapped at all or only with a high risk of misalignment [2]. Such regions pertain to numerous medically relevant genes, such as the drug metabolism gene *CYP2D6,* which has homologous pseudogenes. *SMN1*, a gene that is autosomal recessively linked to spinal muscular atrophy [6], constitutes another example of a clinically relevant but difficult-to-resolve genomic locus since short reads cannot completely discriminate it from *SMN2*. Highly polymorphic regions, including genes of the human leukocyte antigen (HLA) region, are also extremely difficult to map and elucidate with short reads [2,6].

A haplotype refers to an individual set of alleles, i.e., alternative DNA sequences at a specific locus, that has been inherited from a single parent. Sequencing reads can be bioinformatically phased and assembled into individual haplotypes if these reads span at least two heterozygous variants. Haplotype-phased data have several clinical advantages, e.g., the exact determination of the reference alleles or the differentiation between cis and trans compound heterozygous mutations. However, due to the generally low heterozygosity of human genomes [7,8], short reads often do not bridge the distance between two heterozygous single nucleotide variants (SNV) and, therefore, cannot be used for phasing, i.e., assigning variants to a specific haplotype. Taken together, these points significantly limit the ability to correctly identify variants in several important, functional regions of the human genome [1].

The difficulties described above can be resolved by employing longer reads, such as those produced by third generation sequencing techniques [9,10,11]. Oxford Nanopore Technologies (ONT) sequencing currently achieves the longest reads, generating continuous reads of hundreds to thousands of kilobases (kb) in length. However, despite significant advances and recent technical improvements, base calling accuracy is presently not consistently able to match the read quality standards set by Illumina platforms [1,12,13,14]. In addition, extracting phased haplotypes from amplicon-based NGS raw data remains a complex and time-consuming task that requires extensive expertise in bioinformatics.

In contrast to whole genome sequencing (WGS), there is a lack of clearly established software pipelines for processing and analyzing amplicon-based sequencing data to obtain haplotype-specific results. While tools for WGS may, in theory, be coerced to process amplicon-based data, this approach has a major drawback. WGS usually yields limited coverage per base of the reference genome, whereas amplicon-based raw data are able to provide thousands of reads covering the genomic coordinates of a specific amplicon product. Effectively using WGS tools for amplicon-based raw data thus often requires down-sampling or reducing the data depth, which in turn may reduce data quality [15].

Effective data processing of amplicon-based raw data currently necessitates the combination of several publicly available software tools. These individual elements are often poorly documented [16] and require a considerable amount of bioinformatics expertise. In addition, while manual processing of low sample numbers is still feasible, manually processing results from multiple genes and higher sample numbers is not only time-consuming but also error-prone.

At present, there is no automated way to compare and evaluate results from different second and third generation sequencing platforms. We, therefore, developed an automated data analysis framework that allows the processing of amplicon-based, short, and long-read raw data from both ONT and Illumina platforms, combining the strengths of both technologies. The framework is able to automatically generate phased haplotypes and facilitates comparing and evaluating variant information from both Illumina and ONT platforms.

## 2. Materials and Methods

### 2.1. Framework

We constructed a data analysis framework consisting of two principal pathways for processing ONT and Illumina raw data and a final superordinate genotype analyzer module for evaluating and comparing output from both platforms. Each pathway, as well as the superordinate module, can be used independently, as required. The framework is capable of processing and evaluating high-throughput amplicon-based NGS raw data, generating information and phased haplotype assemblies for each sample and platform.

**Figure 1:**
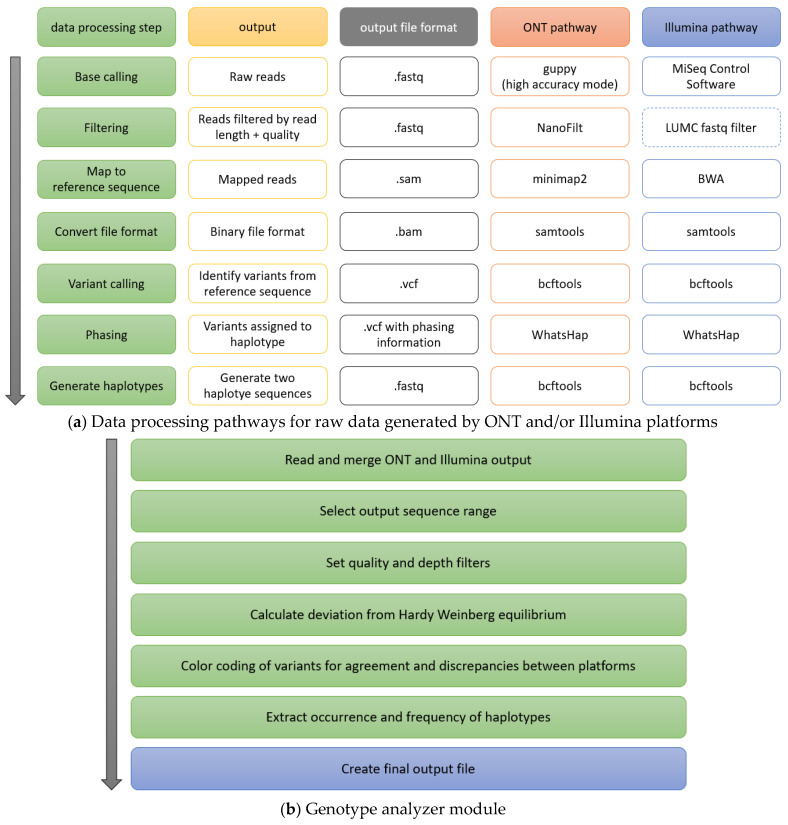
Structure of the data analysis framework. (**a**): Data processing steps of both automated data analysis pathways for massively parallel short-read sequencing raw data generated using Illumina and single-molecule sequencing using ONT platforms. Data from both platforms can be analyzed simultaneously or separately, as required. (**b**): Steps of merging and analyzing output data from both platforms by the genotype analyzer module.

With regard to ONT, the pathway consists of the publicly available tools NanoFilt [17] (version 2.8.0), minimap2 [18] (version 2.24-r1122), samtools [19] (version 1.15, htslib 1.15), bcftools [19] (version 1.15), and WhatsHap [20] (version 1.4). For Illumina, the pathway comprises LUMC fastq-filter [21] (optional; version 1.0.0-dev), BWA [22] (version 0.7.17-r1188), samtools [19] (version 1.15, htslib 1.15), bcftools [19] (version 1.15), and WhatsHap [20] (version 1.4). The structure of the data analysis framework, including a description of each data analysis step, is depicted in Figure 1.

Both pathways automatically yield vcf files with quality and phasing information. Phased haplotypes for each sample and platform are then automatically assembled. All haplotype combinations are counted and listed by frequency. For optimal performance in different applications, quality filters and read length can be adjusted as required.

Haplotype assembly is conducted with WhatsHap, a highly accurate read-based phasing tool primarily designed for third generation sequencing technologies that phases both SNVs as well as indels and other variants. The phaser uses a fixed parameter tractable approach that compares favorably to other state-of-the-art statistical phasers with regard to switch and flip error rates [20,23].

### 2.2. Superordinate Analysis Module

After initial processing of the raw data, the superordinate genotype analyzer module automatically reads, merges, and color codes data from both platforms for agreement and discrepancies. Output variant information includes read depth and quality score for each variant and platform. All variants and haplotype combinations are counted and listed by frequency. Quality control is possible by adjusting read lengths and minimum quality scores. Output variants can also be filtered by read depth and quality score. In addition, deviation from Hardy–Weinberg equilibrium [24] is automatically calculated for each variant position. Further, the output range can be limited to particular areas of interest in the amplicon such as coding sequences or exons only.

### 2.3. Sample Collection and Testing

In order to test the performance of the framework, we designed long-range PCRs for the partly homologous *FUT1*, *FUT2*, and *FUT3* genes that encode the human blood group systems Lewis (ISBT 007) and H (ISBT 018) [25,26,27]. Amplicon lengths were set at approximately 11 kb per gene. Reads were aligned against the reference sequence for *FUT1*, *FUT2*, and *FUT3* of the human genome (build GRCh38; accession numbers NG_007510.2, NG_007511.1, and NG_007482.2). Samples from 400 blood donors from the federal Austrian state of Vorarlberg, Austria, were collected and analyzed as described below. Informed consent was obtained from all study participants.

### 2.4. DNA Extraction and Long-Range PCR

DNA extraction was performed automatically in batches of 96 samples, using standard protocols for the chemagen chemagic magnetic separation module 1 (^®^chemagen Biopolymer-Technology, Baesweiler, Germany). Samples were then frozen and stored at −20 °C.

Long-range PCR was performed separately for *FUT1*, *FUT2*, and *FUT3* on GeneAmp PCR system 9700 (^®^Applied Biosystems, Thermo Fisher Scientific, Waltham, MA, USA) and Sensoquest thermocyclers (^®^SensoQuest, Göttingen, Germany) according to the following protocol. 7 µL of Promega (^®^Promega, Madison, WI, USA) PCR-Master-Mix, 1 µL of 5 µM Metabion (^®^Metabion International, Planegg, Germany) forward primer and 1 µL of 5 µM reverse primer (see Supplementary Table S1), 1.8 µL of betaine^®^ (Sigma Aldrich, Darmstadt, Germany), 5.2 µL of HPLC grade H_2_O, and 2 µL sample with a concentration of 10 ng/µL were amplified. Cyclers were set to start off with 1 cycle of 95 °C for 120 s, followed by 40 cycles consisting of denaturation at 95 °C for 15 s, annealing at 65 °C for 30 s, and elongation at 68 °C for 600 s, ending with a final extension at 68 °C for 600 s. Amplification of target genes was verified by Sanger sequencing on the Hitachi 3730xl DNA analyzer (^®^Applied Biosystems, Thermo Fisher Scientific, USA).

### 2.5. ONT Sequencing

DNA samples of 400 donors were processed in pools of 96 samples according to the ONT (Oxford Nanopore Technologies, Oxford, UK) protocol for amplicon barcoding with native barcoding expansion 96 (EXP-NBD196) and ONT ligation sequencing kit (SQK-LSK109). The resulting libraries were then analyzed on MinION using R9.4.1 flow cells. PCR products of FUT1, FUT2, and FUT3 from each donor were barcoded, pooled, and sequenced. Base calling was performed using Guppy’s high accuracy model (Oxford Nanopore Technologies plc. Version 6.1.3+cc1d765d3) and demultiplexed by barcode ID. An Initial quality check was performed with NanoPlot (version 1.40.0).

### 2.6. Illumina Sequencing

The Illumina NGS library preparation was performed using the NEBNext^®^Ultra™DNA Library Prep Kit for Illumina (New England Biolabs, Ipswich, MA, USA) according to the manufacturer’s instructions. The efficacy of the index PCR was monitored using a SybrGreen-based assay on the GloMax platform (Promega, Madison, WI, USA).

The barcoded fragments were pooled and separated on a 2% TAE agarose gel, and the final library fraction targeting 600–800 bp was cut out and isolated using a GeneJet Gel Extraction Kit (Thermo Fisher Scientific, USA). The eluted library was quantified using a Kapa Library Quantification Kit (Roche, Basel, Switzerland) according to the manufacturer’s recommendations.

Prior to loading the cartridge, the library was diluted to its final concentration of 15 pM following the standard Illumina loading protocol. We performed paired-end sequencing (2 × 250 bp) on an Illumina MiSeq platform (Illumina, San Diego, CA, USA) using a MiSeq Reagent Kit v2 running 500 cycles. Base calling was performed with MiSeq Control Software Version 2.6.2.1.

## 3. Results

### 3.1. Investigated Genetic Regions

For each gene, three different genomic regions of interest were defined and analyzed—coding sequences (CDS), exons, as well as the complete genomic sequence. In order to include potential splice site variants and regulatory element sequences, output ranges were set as follows: CDS plus 50 bp intronic flanking sequences, exons plus 50 bp intronic flanking sequences, and 200 bp of the 5′ and 3′ noncoding regions (‘exons-plus‘), and the full genomic sequence including all exons and introns plus 200 bp of the 5′ and 3′ noncoding regions. The resulting locus lengths for the three target genes are shown in Table 1 for the different target definitions.

### 3.2. Variant Output and Phasing

Based on the third generation ONT sequencing data of all 400 investigated individuals of Caucasian (West-Austrian) origin, the bioinformatic framework identified a total of 357, 2430, and 1244 single nucleotide variants (SNVs) in the CDS of *FUT1*, *FUT2,* and *FUT3*, respectively. Using data generated by the Illumina platform, a total of 352, 2425, and 1210 SNVs were called in the same range. Table 2 gives an overview of heterozygous and homozygous SNVs and insertion–deletion variants (indels) for sequences of the second and third generation sequencing platforms Illumina and ONT.

The accuracy of reported variants was confirmed by comparing a minimum of 15 known variant positions located in the coding sequence of each gene to previously reported variants for the respective rs number. Expected allele frequencies for the European population were extracted from the ALFA NCBI database [28]. Observed allele frequencies were then tested for statistically significant deviation from expected ALFA allele frequencies using chi-square tests. A *p*-value of <0.05 was considered statistically significant. Observed frequencies for each variant position did not differ significantly from expected frequencies for European populations, according to the ALFA NCBI database [29].

The ONT pathway achieved a phasing of 99.7% of Identified variants in the CDS region, 99.2% in the extended exon range, and 98.8% for the complete gene sequence. Illumina allowed for the phasing of 91.9% of identified variants in the CDS region, 87.9% in the exons-plus, and 89.6% of variants in the complete gene sequences.

**Figure 2:**
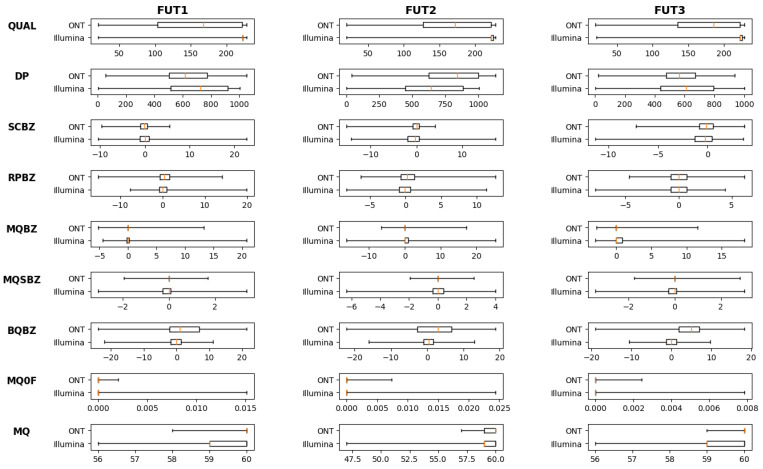
Overall quality score boxplots across complete gene sequences for both ONT and Illumina platforms showcased for *FUT1*, *FUT2*, and *FUT3*. For SCBZ, RPBZ, MQBZ, MQSBZ, BQBZ, and MQ0F, values closer to zero are considered higher quality. QUAL—overall quality score, DP—read depth, SCBZ—Mann–Whitney U-z test of soft-clip length bias, RPBZ—Mann–Whitney U-z test of read position bias, MQBZ—Mann–Whitney U-z test of mapping quality bias, MQSBZ—Mann–Whitney U-z test of mapping quality bias vs. strand bias, BQBZ—Mann–Whitney U-z test of base quality bias, MQ0F—fraction of MQ0 reads, and MQ—average mapping quality.

Quality metrics for the complete gene sequences of *FUT1*, *FUT2*, and *FUT3* from both ONT and Illumina platforms are presented in Figure 2. With regard to clinically relevant coding sequences, average bcftools quality scores and read depths for variants derived from ONT-based data were 165.5 ± 52.7 and 690.5 ± 175.4 for *FUT1*, 167 ± 46.7 and 790.6 ± 237.7 for *FUT2,* as well as 184.7 ± 61.0, and 569.1 ± 131.3 for *FUT3*. Variants identified by the Illumina pathway had average quality scores and read depths of 222.6 ± 1.2 and 880 ± 156.8 for *FUT1*, 223.3 ± 9.9 and 771.9 ± 214.2 for *FUT2*, as well as 223.9 ± 13.9 and 450.1 ± 214.0 for *FUT3*. The quality criteria read depth and QUAL scores were significantly higher for Illumina data compared to ONT (*p* < 0.001 and *p* < 0.001). However, ONT data allowed for the phasing of a higher percentage of identified variants.

### 3.3. Agreement Between Second and Third Generation Platforms

Variant information from both ONT and Illumina platforms was automatically merged, compared, and evaluated by the superordinate genotype analyzer module. In the resulting output file, each variant was color-coded for agreement and discrepancies between the two platforms. Green was used to designate agreement between both platforms, red for discrepant results, yellow if a variant was reported by the Illumina platform but not ONT, and orange if a variant was reported by the ONT platform but not by Illumina. To facilitate identifying individual samples or variant positions of low data quality, the frequencies of each case were reported for each variant position and sample.

**Figure 3:**
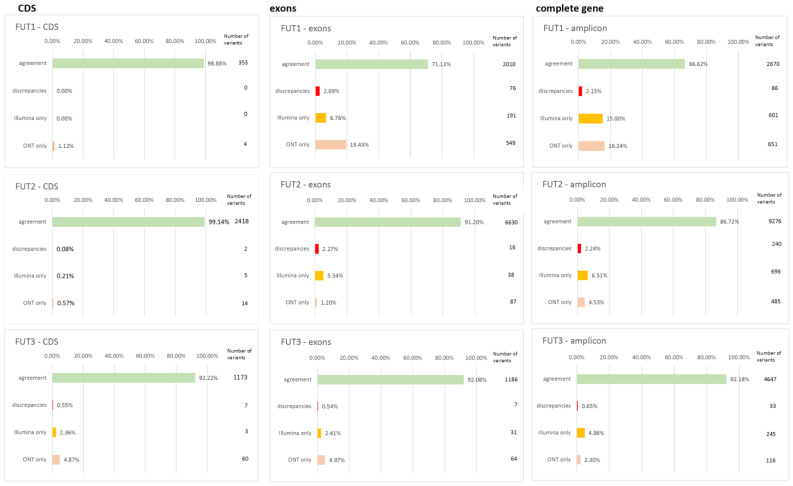
Agreement and discrepancies (%) in variant information between ONT and Illumina platforms by coding sequences (CDS), ‘exons-plus’, and the complete genomic gene sequences for *FUT1*, *FUT2*, and *FUT3*. Green bar—agreement, red bar—discrepant information, yellow bar—variant reported by Illumina but not ONT, and orange bar—variant reported by ONT but not Illumina.

**Figure 4:**
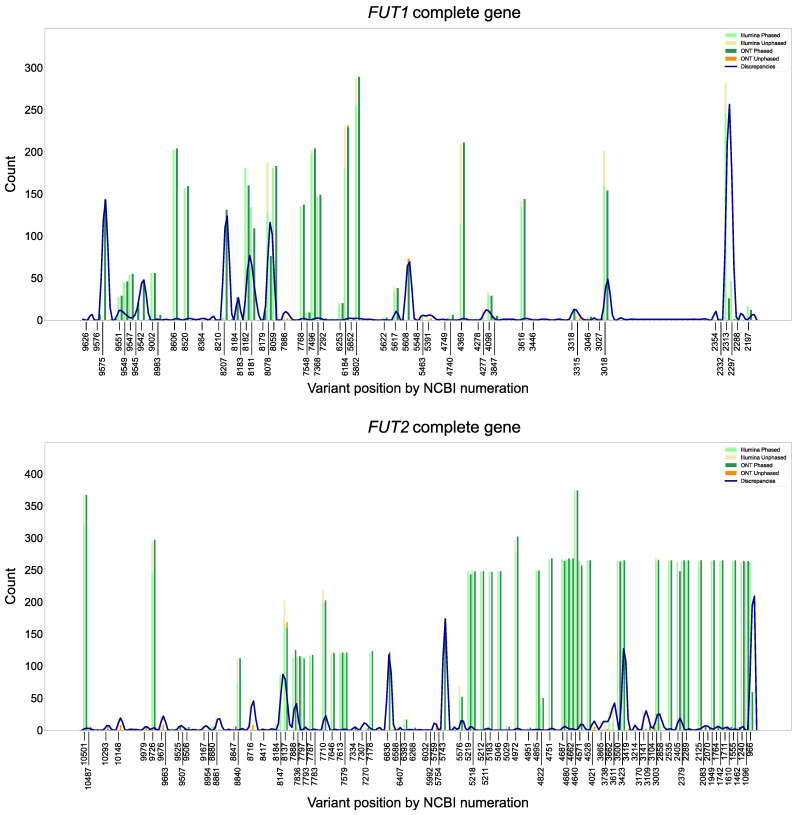

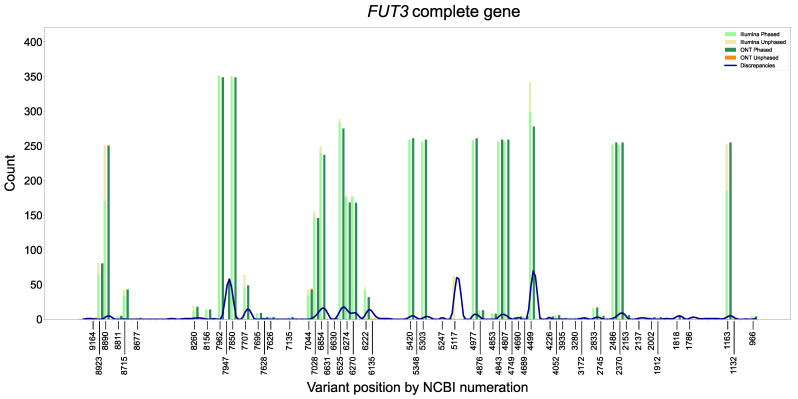
Number of variants and discrepancies between ONT and Illumina platforms per variant position and *FUT1*, *FUT2*, and *FUT3* gene. The number of both phased and unphased positions for Illumina and ONT is displayed. Blue line: number of discrepancies between the two platforms. ONT—Oxford Nanopore Technologies.

Agreement in variant calling between ONT and Illumina systems across coding sequences was 98.9% for *FUT1*, 99.1% for *FUT2*, and 92.2% for *FUT3*. Detailed results for agreement and discrepancies in variant information between ONT and Illumina platforms for CDS, exons, and gene sequences are illustrated in Figure 3. In addition, the number of detected variants and discrepancies between ONT and Illumina per variant position and gene is depicted in Figure 4.

### 3.4. Performance

Next, we compared time requirements for manual, semi-automated, and fully automated data processing approaches by running simulations for the analysis of *FUT1*, *FUT2*, and *FUT3* sequences derived from ONT and Illumina platforms in samples of 400 blood donors. The manual approach was defined as manually inputting each command for each sample and gene in the Linux console window. Manually executing each software tool but automating the process for all samples was considered a semi-automated approach. Both manual and semi-automated methods require expertise in Linux console commands. In addition, the semi-automated approach also requires proficiency in automation tools such as bash script. The fully automated approach was conducted by the framework described above.

**Figure 5:**
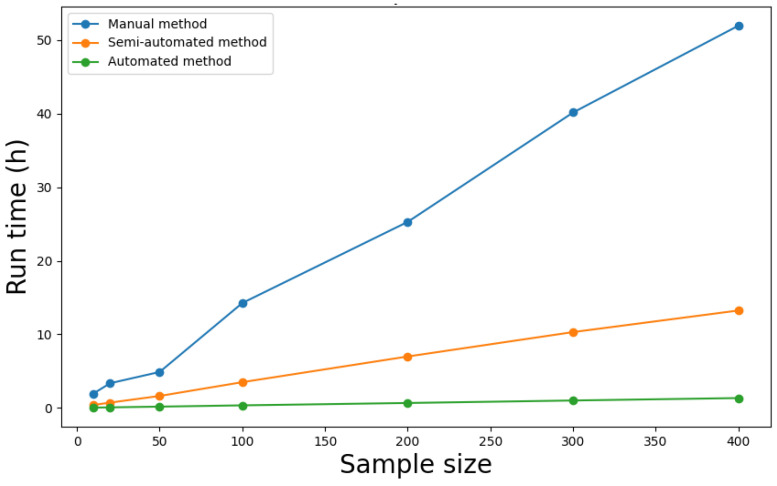
Comparative assessment of the time requirements for manual, semi-automated, and fully automated processing of next-generation sequencing (NGS) raw data generated from Oxford Nanopore Technologies (ONT) and Illumina platforms, based on three genes (~30,000 base pairs in total) across 400 blood donor samples. Additional time requirements for manual merging and comparison of ONT and Illumina output are not represented.

The simulations only encompassed the processing of raw NGS data but not the steps required for merging, comparing, and evaluating data from both platforms that are performed by the genotype analyzer module. The calculation process also did not incorporate subsequent data analysis steps, such as determining haplotype and variant frequencies across all samples. The results are shown in Figure 5. All simulations were run using the following system: 11th Gen Intel^®^ Core™ i9-11950H 2.60 GHz (Intel Corporation, 2200 Mission College Blvd., Santa Clara, CA 95054, USA), 32 GB DDR4 RAM 3200 MHz, NVIDIA RTX A4000 (NVIDIA Corporation, 2788 San Tomas Expressway, Santa Clara, CA 95051, USA), operating system Win10 Pro (Microsoft Corporation, One Microsoft Way, Redmond, WA 98052, USA).

### 3.5. Synthetic Data Framework Validation

In order to verify the validity of the data analysis framework, we generated synthetic data reads with and without SNVs for both ONT and Illumina platforms in three different quality levels and analyzed them with the previously presented framework. Naturally occurring variants account for approximately one SNV in every 800 to 1000 bp [30]. As our data are derived from amplicons with an approximate length of 10,000 bp per *FUT* gene, we simulated a total of 10 variants per gene. Synthetic reads were generated with ART (ART-GreatSmokyMountains-04-17-2016 v2.5) [31] for Illumina reads, using the HiSeqX algorithm, the HS25, and the HS10 algorithms to simulate high, moderate, and low data quality. Pbsim3 (v.3.0.1) [32] was used to simulate reads generated by an ONT platform. Accuracy was set to 87–89%, 92.5–94.5%, and 96–98% to simulate low, moderate, and high-quality reads. In all simulations, each introduced SNV was correctly identified, with the exception of one SNV (0.03%) that was misidentified as heterozygous instead of homozygous by the ONT platform in the lowest quality setting. No additional SNVs were reported by the framework. Validation data are available in Supplementary File S1.

## 4. Discussion

We constructed a modular data analysis framework consisting of two principal pathways for processing ONT and Illumina raw data and a final superordinate module for integrating, evaluating, and comparing output from both platforms. Each pathway, as well as the superordinate module, can be used independently according to individual requirements.

### 4.1. Key Results

The framework is capable of processing and evaluating high-throughput amplicon-derived NGS raw data, generating variant information and phased haplotypes for each sample and platform.

In addition, the framework includes various options to control and adjust the NGS data quality. For instance, filters for quality scores and read lengths can be adjusted as required [33,34]. Sequence output ranges can be restricted to particular genomic regions of interest, such as CDS or exons only. Quality scores and read depths are also reported for each variant. Further, the color coding of variants for agreement between Illumina and ONT platforms facilitates the identification of variant positions or individual samples with high rates of discrepancies between the two platforms, thereby highlighting variant positions, genomic regions, or samples that require close scrutiny. For each reported variant position, a calculation of the Hardy–Weinberg equilibrium [35,36] is performed, allowing the identification of potential, albeit unexpected, deviations.

All software tools employed by the presented data analysis framework for processing NGS raw data are freely available. The modules for automatically processing both ONT- and Illumina-derived data, as well as the superordinate genotype analyzer module described above, are also freely available.

### 4.2. Interpretation

The Illumina platform produces highly accurate sequencing reads (>99.9%) and has, therefore, become the gold standard of clinical and research sequencing [1]. However, Illumina typically produces short reads of <300 bases, which has several important limitations.

First, due to the low heterozygosity of human genomes [7,8], these short reads often only cover a single variant site and, therefore, cannot provide phasing information for haplotype building.

Second, around 2.2% of exons from medically relevant genes cannot be uniquely mapped with short reads because identical matches exist elsewhere in the genome. A further 3.9% of exons are highly homologous and thus carry a high risk of misalignment. This issue concerns regions related to numerous disease-associated genes, such as *SMN1* (spinal muscular atrophy) and *CFC1* (congenital heart defects) [6].

Third, the reads provided by Illumina are too short to reliably detect >70% of human genome structural variations, especially those of intermediate size, because many of the structural variant calls map to tandem repeats that cannot be spanned by short reads [1,2,37].

Fourth, amplification steps during library preparation and/or during the sequencing reaction may introduce chimeric reads that hamper read alignment and may diminish the quality of the downstream analysis [2].

Fifth, short-read technologies are prone to GC bias, an underrepresentation of sequences with atypical GC content, i.e., GC-rich or GC-poor regions [2,38].

Sixth, correct alignment of reads is particularly challenging in highly polymorphic regions, such as the major histocompatibility complex (MHC) or HLA region, in particular *HLA-B*, because the number of discrepancies between reference and analyzed sequence increases the risk for misalignment [2,33]. These difficulties can be resolved by using longer reads, such as those generated by ONT single-molecule sequencing.

While the dual approach of the framework profits from the high accuracy of the Illumina reads, less accurate but long ONT reads contribute to resolving the previously described drawbacks of short-read sequencing. ONT currently provides the longest sequencing reads, generating continuous reads of hundreds to thousands of kilobases in length. This affords an important advantage of correctly mapping reads to homologous regions, spanning repetitive regions, detecting structural variation, covering sequences with atypical GC contents, and phasing distant variants separated by hundreds of base pairs [1]. Thus, the framework leverages the best of two methodological approaches to maximize reliability.

This framework may also be used in combination with adaptive sampling, an ONT approach for software-controlled enrichment of target sequences, in which sequencing of unwanted, off-target fragments can be aborted by ejection of the molecule from the pore [39]. This approach allows for an approximately 5-fold enrichment without the necessity of a PCR or laboratory workflow adaptation [39]. In combination with amplicon-based sequencing, ONT adaptive sampling may thus provide a further increase in sensitivity. However, due to the already high coverage achieved by amplicon-based sequencing, the added benefit may be marginal. In contrast, WGS with ONT adaptive sampling would still yield substantially lower coverage than the amplicon-based sequencing for which this framework was designed. Adaptive sampling alone may, therefore, be disadvantageous compared to direct amplicon sequencing with both Illumina and ONT.

The presented framework has been designed specifically in order to combine the long reads offered by ONT with the higher accuracy offered by Illumina. The aim of this approach was to allow for the phasing of reads that could not be phased by short-read technologies alone while compensating for the lower accuracy of the ONT platform. While the PacBio platform offers higher accuracy [1], we chose ONT for several key reasons. Firstly, ONT platforms are more widely accessible and scalable for laboratories with varying resource levels. ONT also allows flexible, rapid sequencing with shorter turnaround times, which is particularly advantageous in clinical or high-throughput settings [1]. Secondly, ONT sequencing remains significantly more cost-effective per run, especially when processing hundreds of samples. In contrast, PacBio HiFi sequencing is more expensive, which could limit the scalability and the generalizability of our framework for larger studies [1,40]. Thirdly, this framework aims to combine the best of two fundamentally distinct sequencing technologies while alleviating their weaknesses. Replacing ONT with PacBio, which has closer accuracy levels to Illumina [1], would reduce the contrast and potentially the utility of our comparison. Nonetheless, PacBio offers valuable advantages and may be well suited for future studies.

Another approach that has been employed to overcome some of the limitations of short-read technologies is the generation of synthetic long reads. However, in comparison to true long-read sequencing, several drawbacks need to be taken into account [1,40]. Firstly, the assembly process may be prone to errors, especially in low-complexity or repetitive regions. Secondly, reconstructing long reads from many overlapping short reads requires significant computational resources and sophisticated algorithms. Thirdly, although synthetic long reads can span several kilobases, they are still generally shorter than native long reads from long-read platforms. Fourthly, in regions with high sequence complexity, synthetic long reads may fail to assemble accurately or may not reach the necessary length. Synthetic long reads may further not perform well in detecting structural variants, phasing haplotypes, or analyzing highly repetitive or GC-rich regions compared to true long-read sequencing.

*FUT1*, *FUT2,* and *FUT3* define the clinically relevant human blood group systems H and Lewis. In particular, variations in both *FUT2* and *FUT3* have been associated with various types of disease including different types of cancer and intestinal diseases [41]. Homologous sequences continue to pose major challenges for short-read technologies, potentially leading to false positive and false negative diagnostic errors [6]. For the partly homologous genes *FUT2* and *FUT3*, a significant divergence between phenotyping and genotyping results has been reported [42]. We, therefore, chose to focus on these challenging areas to test the performance of the data analysis framework described above.

Discrepancies between ONT and Illumina occurred primarily in regions with repetitive sequences of AT, poly-A sequences, and high GC content. This is in accordance with previous studies that have shown aberrant results for Illumina in regions with atypical GC content [2,38] and for ONT in regions with low complexity due to repetitive sequences, as there are only minor variations in the electric signal of the pore if the base does not change [43].

### 4.3. Performance

While the actual time needed for each method is highly dependent on the data volume and the computer systems used, we nevertheless deem the proportions between manual, semi-automated, and fully automated approaches to be a good representation of the relative time required for each method. Both the manual and semi-automated approaches require extensive expertise in bioinformatics, preinstalled software tools, and a predefined sequence of console commands. Manually comparing variant output from both ONT and Illumina platforms was not included in the calculations but would require considerable time and effort depending on sample numbers and gene sequence lengths.

### 4.4. Limitations

This framework was designed for processing and analyzing amplicon-based second and third generation sequencing data and, therefore, has limited applicability for whole genome sequencing. While this framework allows for processing and comparing data generated by second and third generation sequencing platforms, agreement between these methodological approaches will depend on library quality and on the characteristics of the targeted genomic region, in particular with regard to highly polymorphic regions, the presence of homologous or highly repetitive sequences, or atypical GC content. As a consequence, while we provide various options for filtering and quality control of sequencing data, settings may have to be adapted depending on the targeted genomic region and the quality of the available data.

We deliberately refrained from automatically reconciling discrepancies between both platforms because a generic, one-size-fits-all solution carries a substantial risk of introducing false positives or false negatives (suppressing true variants or accepting incorrect calls). Given the distinct error profiles and platform-specific biases [1,2,38], a number of factors may result in incorrect conflict resolution if discrepancies are automatically reconciled without contextual awareness.

Firstly, the characteristics of the analyzed genomic region, including but not limited to GC content, short tandem repeats, and homopolymer regions, may disproportionately affect the error rate of one platform over the other [2,38]. Depending on the sequence in question, a uniform, automated solution for reconciling discrepancies may, therefore, result in a high number of misclassifications.

Secondly, variations in run quality may be considerable and may also result in increased error rates in the case of automated reconciliation. The presented framework allows setting minimum quality requirements to mitigate this effect but human oversight may still be required.

Thirdly, while phasing information is expected to be more consistent in ONT-derived data due to the longer reads [1], a potential error in variant calling may offset this advantage. Given the high coverage provided by both platforms in this approach, discrepancies occurring in amplicon-based sequencing need to be examined in detail to avoid misclassifications.

Future studies are warranted to assess whether AI or machine learning approaches may be useful to overcome some of these difficulties. However, although promising, such models may still yield substantial error rates when used without human oversight, particularly in complex genomic regions.

Importantly, this framework is built to be extensible. Thus, user-defined reconciliation strategies tailored to specific requirements may be incorporated in order to automatically reconcile discrepancies where feasible. In addition, the framework offers tools to detect patterns of discrepancies across large datasets, streamlining the identification of systematic biases or errors.

### 4.5. Comparison to Other Tools

To the best of our knowledge, there is no directly comparable software for combining amplicon-based data from two distinct sequencing platforms and leveraging the best of both worlds for an informed decision on genomic variation.

While there are now various pipelines for whole genome sequencing available [44], few options exist for the more specialized demands of amplicon-based sequencing, which are able to produce significantly higher per base coverage with up to thousands of reads. Single tools for each step have been provided, but no clearly established pipeline for processing both Illumina and ONT amplicon-based raw data has been described.

Another approach is polishing relatively noisy ONT reads with Illumina reads for the purpose of, e.g., de novo genome assembly [45]. The disadvantage of this approach is that the user cannot directly compare the information originating from both technologies to make informed decisions. Further, this approach is prone to a variety of errors, including biased correction, the potential introduction of bias by Illumina reads, which may lead to loss of information present in ONT reads [12], error propagation where incorrect Illumina reads may propagate errors into ONT reads that lead to inaccuracies in the downstream analysis [46], and potential loss of long-range structural information [47].

Hence, our framework is the first software that allows sophisticated interpretation and combination of second and third generation methods in the case of amplicon sequencing, which is especially relevant for smaller laboratories. The framework presented here may also be extended to provide data processing and evaluation for additional second and third generation sequencing platforms.

## 5. Conclusions

This open-access, modular data analysis framework allows for simple, flexible, and time-efficient processing and analysis of amplicon-based NGS raw data generated by both Illumina and ONT systems. The framework is capable of automatically comparing results, highlighting discrepancies, determining haplotypes, and listing haplotype frequencies. Output from both systems is merged and evaluated, combining the strengths of both technologies.

## Figures and Tables

**Table 1:** Different target definitions for the target genes *FUT1*, *FUT2*, and *FUT3*.

	Target Genes		
Definition	FUT1 [bp]	FUT2 [bp]	FUT3 [bp]
CDS	1197	1132	1185
exons-plus	5022	3614	3162
complete gene	7747	10,380	8961

**Table 2:** Number of total reported variants (SNVs and indels), heterozygous variants, and percentage of phased variants per complete genomic sequence and amplicon region are shown. Results are based on raw data from both ONT and Illumina platforms from analyzing samples of 400 blood donors. CDS coding sequence and SNV single nucleotide variant.

	CDS		Exons-Plus		Complete Gene	
	ONT	Illumina	ONT	Illumina	ONT	Illumina
*FUT1*						
# SNVs (all/heterozygous)	357/308	352/304	2614/2172	2251/1944	3377/2780	3316/2872
# indels (all/heterozygous)	0/0	0/0	21/18	17/16	38/32	29/28
% phased	100	98.7	99.1	87.7	98.5	86.7
*FUT2*						
# SNVs (all/heterozygous)	2430/1835	2425/1830	6836/4752	7101/5098	9873/7110	9970/7331
# indels (all/heterozygous)	4/0	0/0	46/21	82/81	128/81	242/240
% phased	100	98.1	99.5	97.0	98.6	92.8
*FUT3*						
# SNVs (all/heterozygous)	1244/660	1210/648	1257/673	1224/662	4787/3276	4856/3412
# indels (all/heterozygous)	0/0	0/0	2/2	0/0	11/11	69/68
% phased	99.2	79.0	99.0	79.0	99.4	89.1

## Data Availability

Sequencing data from both Illumina and ONT platforms will be made available at the European Genome-Phenome Archive on https://ega-archive.org/ (accessed on 6 May 2024). The data analysis framework and software code are publicly available on https://github.com/ChrAtt1/Sequencing_Data_Analysis_Framework (accessed on 3 September 2024).

## Supplementary Materials

The supporting information can be downloaded at: https://www.mdpi.com/article/10.3390/ijms26073443/s1.

## Abbreviations

NGS	next-generation sequencing
ONT	Oxford Nanopore Technologies
WGS	whole genome sequencing
PCR	polymerase chain reaction
