# Supplementary material for: Merging High-Throughput, Amplicon-Based Second and Third Generation Sequencing Data: An Integrative and Modular Data Analysis Framework for Haplotype Prediction and Output Evaluation
Source: Int J Mol Sci. 2025 Apr 7;26(7):3443. doi: 10.3390/ijms26073443 (PMC11990026; doi:10.3390/ijms26073443)
Supplement: Supplementary file 1 [file ijms-26-03443-s001.zip › Supplementary table S1.pdf]

Supplementary material

**identifier**	**gene**	**primer type**	**bases**	**5'-3' sequence**
FUT1-3123-F2	*FUT1*	forward	23	CTCCAGCATCCACACATCACATG
FUT1-12893-R2	*FUT1*	reverse	24	TTGTTGCTGGACTTGTGTGAGTTG
FUT2-7256-F1	*FUT2*	forward	25	CTGCCTGGAGGAGCGGAGCAGAGTG
FUT2-3368-R	*FUT2*	reverse	26	CTATTACAAGTCTGGATCAAAAAGGC
FUT3-7416-F	*FUT3*	forward	30	AAGGGCTGGAGGGGGAGAATATTACAAAGT
FUT3-3674-R2	*FUT3*	reverse	26	GCCCAGCGATCACATCGATGGAATGA

Supplementary table S1. Primer sequences for long range PCR amplification of *FUT1*, *FUT2* and *FUT3.*
